# Short Road Transport and Slaughter Stress Affects the Expression Profile of Serotonin Receptors, Adrenocortical, and Hematochemical Responses in Horses

**DOI:** 10.3390/vetsci11030113

**Published:** 2024-03-03

**Authors:** Giuseppe Bruschetta, Gabriella Zanghì, Renato Paolo Giunta, Alida Maria Ferlazzo, Katiuska Satué, Angela D’Ascola, Esterina Fazio

**Affiliations:** 1 Department of Veterinary Sciences, University of Messina, Via Palatucci Annunziata, 98168 Messina, Italy; alida.ferlazzo@unime.it (A.M.F.); esterina.fazio@unime.it (E.F.); 2 Department of Catania, Experimental Zooprophylactic Institute of Sicily, Via Passo Gravina 195, 95125 Catania, Italy; gabriella.zanghi@hotmail.com (G.Z.); renato.giunta@izssicilia.it (R.P.G.); 3 Department of Animal Medicine and Surgery, Faculty of Veterinary Medicine, CEU-Cardenal Herrera University, Tirant lo Blanc 7, Alfara del Patriarca, 46115 Valencia, Spain; ksatue@uchceu.es; 4 Department of Clinical and Experimental Medicine, Policlinico Universitario, University of Messina, Via Consolare Valeria 1, 98125 Messina, Italy; adascola@unime.it

**Keywords:** cortisol, 5-HT receptors, horse, serotonin, slaughter, transport stress

## Abstract

**Simple Summary:**

The aim of this study was to evaluate the circulating serotonin concentration and the pattern of serotonin-related receptors (5-HT1B and 5-HT2A) expressed by peripheral blood mononuclear cells (PBMCs), and the adrenocortical and hematochemical responses of horses exposed to transportation and slaughter stress. We found that the 5-HT1B and 5-HT2A expression levels were significantly increased by transportation and slaughter stress, as well as several stress parameters, while serotonin’s concentration was markedly reduced. Based on this evidence, the evaluation of serotoninergic system alterations may be useful to better explain the impact of transportation and slaughter on horse welfare, with a potential application for improving the protocols and guidelines for transported horses.

**Abstract:**

Horse transport is considered a cause of stress in animals and is known to affect the 5-HT concentrations in both the brain and other tissues. The aim of this research was to investigate the effect of horse transportation and slaughter stress on plasma serotonin’s concentration and the expression levels of the related 5-HT1B and 5-HT2A receptors in PBMCs. Furthermore, the IL-12 levels and a variety of blood parameters, including triglycerides, total cholesterol, glucose, aspartate aminotransferase, creatine phosphokinase, lactate dehydrogenase, were also considered. This research was carried out on 32 horses submitted to short road transport of 40 km to slaughter. Blood samples were collected in baseline conditions (T0) and 24 h later, after they were slaughtered (T1). The results showed a significantly increased expression of 5-HT1B and 5-HT2A receptors and a significantly decreased expression of IL-12 in PBMCs at T1 vs. T0. Furthermore, a significant increase in cortisol and glucose concentrations, and LDH activity was observed at T1. In contrast, a significantly lower circulating 5-HT concentration was observed at T1 vs. T0. These results indicate that the stress induced by transport and slaughter stimuli led to the serotoninergic system’s activation, suggesting that the expression of serotonin receptors could be used as a pivotal marker of stress, with potential applications for the improvement of elective protocols to observe the guidelines relating to transported horses.

## 1. Introduction

Transport is a stressful routine procedure, representing a pivotal source of physical and psychological stimuli to animals’ homeostasis [1,2,3,4]. The hypothalamic–pituitary–adrenal (HPA) axis response and blood parameters represent the most reliable system used to investigate stressful conditions [5], immune function, and energy storage according to the expenditure in the organism; the pathway of this axis results in the synthesis of cortisol [6].

In addition, serotonin (5-HT) represents an immunomodulatory biogenic amine, which covers neurotransmitter and stress-response mediator functions [7]. 5-HT is indeed involved in physiological and behavioral responses, regulating various immunomodulatory effects [8]. There is evidence that high 5-HT concentrations seem to stabilize the HPA axis’ response and low concentrations appear to facilitate or induce stress and/or aggressive responses [9,10,11]. Some studies highlight a link between deficits in the serotonergic nervous system and the HPA axis’ hyperactivity [12]. Furthermore, 5-HT also represents a sensitive modulator of both physiological and pathological effects of stress [13,14,15]. Severe and acute diseases are linked with the lowest serotonin concentrations in the horse [16], while the highest 5-HT concentrations were measured in horses affected by chronic disease or short surgery, as well as by physical exercise [17,18,19,20,21].

The peculiarity of the serotoninergic system involved in the stress responses is represented by the multiple number of 5-HT receptors placed in both pre- and post-synaptic sites [14]. Some receptors, but not all, show a specific sensitivity to stressors [22]. Indeed, stress induced modifications in the sensitivity of the somatodendritic 5-HT1A autoreceptor, but not in their numbers, in the rat dorsal raphe nucleus [23]; in addition, acute stress can either induce increases or decreases in hippocampal and/or cortical 5-HT1A receptors [24]. 5-HT1A, 5-HT2A, 5-HT1B, and 5-HT7 receptors are significantly represented in leukocytes [25], and it is well established that the serotoninergic system may also induce the regulation of cytokine production [25]. Platelets are the main 5-HT reserve in the peripheral tissues, releasing 5-HT in a superimposed manner to central serotoninergic synaptosomes [26], and guaranteeing low plasma 5-HT concentrations. 5-HT is often stored in neurons and enterochromaffin cells as a co-transmitter.

Furthermore, the crosstalk between the energy metabolism in a catabolic or anabolic way after stressful exposure is also characterized by significant hematochemical changes that normally mirror the animals’ physiological state, according to their homeostasis and/or imbalances [27,28,29].

Transport stress before slaughter, and slaughter may contribute to the disruption of animals’ homeostasis and welfare, and, consequently, their meat quality. On these bases, the hypothesis of the present research was that the physiological response to stressful stimuli could significantly involve the activation of both the HPA axis and the serotoninergic system, presumably providing a frontline of defense against threats to homeostasis.

Based on the increasing attention on animal welfare during their transport, and the effects of their status on the meat quality for human consumption, the aim of this research was to compare horses’ responses in baseline conditions (T0) and when subjected to short road transport and slaughter (T1) to confirm or deny whether these procedures could induce an aversive effect on horses’ adaptive responses.

## 2. Materials and Methods

### 2.1. Ethical Approval

All animal care, treatments and routine procedures followed the guidelines of the EU Directive 2010/63/EU, the Italian law D.L. 26/2014 for ethical care and use of experimental animals, the UE Directive 86/609/CEE, and the regulation (EC) 1/2005 on the protection of animals along transport and relative operations.

All analyses were carried out on animals to be slaughtered for human consumption, in accordance with the Italian and European reference legislation, which guarantees animal protection at slaughter (EU Directive 93/119/EC). The Committee for the Care and Use of Animals of Messina University declared that the proposed study could not be considered an animal experimentation and thus did not need ethical approval according to Italian law. Informed consent from the horses’ owners was obtained for each animal that participated in this study.

### 2.2. Animals and Blood Sample Collection

This study was performed on 32 healthy Franches-Montagnes and cross-bred farmed horses used for meat production (10 stallions, 6 geldings and 16 mares), aged 6 ± 3 years, weighing 550–600 kg. Each horse was taken from an individual stall, loaded simultaneous using 6 transport vehicles, 5 of which were loaded with a maximum of 6 horses, and 1 vehicle with a load of the last 2 horses, and transferred from its stud farm to slaughter, from Catania, Sicily, Italy (37°34′00″ N, 14°54′09″ E) to a commercial slaughterhouse, for a total of 40 km. All subjects did not have previous experience or familiarity with trailer and transport procedures, caretaker, and co-specifics. Water and hay were not available during transport.

Blood samples (10 mL) were collected by jugular venipuncture in evacuated tubes with serum or with heparin (Venoject^®^, Terumo, Leuven, Belgium) at 09:00 a.m. in each subject’s box 24 h before loading and transportation in baseline conditions (control, sample T0). The day after, the horses were transported to the slaughterhouse, and the second blood sample was taken immediately after they were slaughtered (sample T1). Blood sampling took a few minutes for each subject. Blood samples were centrifuged at 3000× *g* rpm for 20 min in order to separate the serum that was stored at −20 °C until the assay of cortisol and the hematochemical analyses. Platelet-poor plasma (PPP) was obtained by centrifugation, at 4500× *g* for 10 min at 4 °C, using evacuated tubes containing heparin [17] and the supernatants were stored at −80 °C, for a week, until the analysis of 5-HT. Peripheral blood mononuclear cells (PBMCs) were separated from whole blood for the evaluation of 5HT1B and 5HT2A receptors, and of IL-12 expression by RTqPCR.

Inclusion criteria for the study were that all horses selected had to be healthy and in good body conditions, without infectious diseases and injury, including lameness, based on the examination by the veterinary doctor before slaughter. During the period of lairage (1 h), the animals did not have free access to feed, only to water, and were handled carefully to avoid harm or injury. Subsequently, the horses were moved to the stunning box, and stunned using a penetrating captive bolt pistol impelled by air and bled by jugular vein sticking. After stunning with one shot, the horses showed paddling movements with their legs. A time of 90 **s** represented the maximum stun-to-stick interval. In order to verify correct stunning had been carried out, the following signs were analyzed: the animal immediately fell and did not try to stand up; respiratory arrest occurred; the muscles of the animal immediately became rigid after the shot; and the eyelid was open with the eyeball facing straight ahead and not rotated.

### 2.3. Transport Vehicle and Environmental Conditions

The commercial trailer used for transport was 9.5 m long and 2.5 m wide, with a ceiling height of 2.5 m (Eurocargo Iveco, Iveco S.p.A., Turin, Italy). The horses were separated in six compartments with swinging gates. The vehicle was equipped with partitions, which allowed six horses to be loaded in a perpendicular configuration to the direction of travel. Six single compartments with swinging gates were available, with a total of 6 horses per load for 5 trips, and 2 horses for 1 trip. The stocking density was about 2 m^2^/horse (6 horses/load), and rubber padding lined the sides of the trailers from the floor up to an approximate height of 1.3 m. The number of horses per load, the floor area available, distance travelled, and the time between loading and unloading were recorded. Feed and water were provided before loading but not during transportation. The animals were randomly orientated, and per each load, the horses were placed perpendicular to the direction of travel. A total of 5 trips were made, with a full load of 6 horses for 5 trips, and 1 trip with only 2 horsepower. All trips were performed on the same day and at the same time, and the departure location was the same stud farm for all horses. Specifically, the horses were transported for 40 km, and the trunk road duration was of a period under 1 h, at an average speed of about 60 km/h. The transport procedure took place in April with a temperature of 18 °C with 62% of relative humidity. The temperature and relative humidity inside the trailer were continually monitored using Hygrothermograph ST-50 (Sekonic Corporation, Tokyo, Japan). At the beginning of the trip, the values were, respectively, 18 °C and 62.4%, and upon arrival at the slaughterhouse (1 h later), these were, respectively, 20.4 °C and 63.5%.

### 2.4. Cortisol and 5-HT Analyses

Serum cortisol was measured in duplicate by a commercial competitive enzyme immunoassay (EIA, RADIM, Rome, Italy), and a BRIO automated analyzer (SEAC, Rome, Italy). Cortisol samples were incubated with cortisol conjugated to horse radish peroxidase (HRP) that competed for the peculiar sites of the antiserum coated on the wells. Subsequently, each well was aspirated and washed three times. A substrate chromogen solution (tetramethylbenzidine, TMB) was added in order to measure the enzyme activity bound to the coated wells that was inversely correlated to the cortisol concentration both in the calibrators and the samples. The plate was incubated for 30 min at room temperature, and optical density (OD) was measured at 450 nm, 405 nm wavelength using a spectrophotometer (Sirio S, SEAC, Florence, Italy). Immunoassay sensitivity was paired to 13.80 nmol/L. The average intra- and inter-assay coefficients of variation were, respectively, paired to 4.0% and 6.9%.

The plasma 5-HT concentration was measured in PPP fraction using an ELISA Assay kit (BioVision incorporated, Milpitas, CA, USA) according to the manufacturer’s protocol. Briefly, 50 μL of standard or sample was added to each well, mixing with 50 μL of biotin-detection antibody working solution. After incubation for 45 min at 37 °C, the wells were aspirated and washed three times. Subsequently, 0.1 mL of SABC working solution was added into each well and the plate was incubated at 37 °C for 30 min. After washing, 90 μL of TMB substrate was added into each well and the plate was incubated at 37 °C for 15–30 min before the stop solution’s addition. The OD values were read at 450 nm (A560, Fulltech, Rome, Italy). The 5-HT concentration of the samples was obtained by interpolation from the standard curve concentration values. The results were expressed in ng/mL. The sensitivity of the assay was 0.5 ng/mL. The average intra- and inter-assay coefficients of variation were, respectively, paired to 5.2% and 9.1%.

### 2.5. Peripheral Blood Mononuclear Cells’ (PBMC) Isolation, RNA Extraction and Reverse Transcription

Blood samples were mixed with a phosphate-buffered saline (PBS) solution (1:3), and put onto a volume of Lymphoprep (Cedarline, Surrey, BC, Canada) for density centrifugation at 350× *g* for 30 min at room temperature. After layering, the buffy coat containing peripheral blood mononuclear cells (PBMC) was collected and washed with PBS at 300× *g* for 10 min. The washing step was repeated three times at 300× *g* to remove contaminating platelets in the PBMC. Finally, the cells were harvested for RNA isolation and cDNA synthesis. For the isolation of total RNA from PBMCs, 1 × 10^7^ cells were harvested and mixed with Buffer RLT. After the lysis step, all samples were centrifuged at 12,000× *g* for 3 min and the supernatants were transferred to new tubes before the addition of an equal volume of 70% ethanol. After mixing, 700 μL of the samples was transferred to an RNeasy Mini spin column centrifuged for 15 s at 8000× *g*. Subsequently, the flow-through was discarded and the columns were washed. Finally, 50 μL of elution buffer was added to the spin columns and the tubes were centrifuged for 1 min at 8000× *g* to elute the RNA. Total RNA (2 μg) was converted into cDNA by High-Capacity cDNA Reverse Transcription Kit (Themo Fischer scientific, Waltham, MA, USA) in accordance with the manufacturer’s protocol.

### 2.6. Real-Time Quantitative PCR (qPCR)

RT-PCR reactions were performed using specific TaqMan Assays and the TaqMan Universal PCR Master Mix (Applied Biosystems, Foster City, CA, USA) to evaluate the expression of IL-12, 5-HT1B and 5-HT2A receptors. β-actin was used as the endogenous control, and the reactions were performed using the Real-Time PCR system model 7500 (Applied Biosystems, USA). The expression of target genes was quantified by calculating the cycle thresholds (CT) and normalizing against β-actin. A relative quantification was performed using the 2^−ΔΔCt^ method. For the analysis in PBMCs, the calibrator was chosen as the mean value of the target genes’ levels of samples taken from the horses before the transfer to the slaughterhouse (T0). The results were expressed as relative fold changes.

### 2.7. Analysis of Hematochemical Parameters

Hematochemical analyses were performed within 3 h of serum sample collection by a spectrophotometer UV-Vis (Sirio S, SEAC, Florence, Italy). Glucose, total cholesterol (TCHOL), triglycerides (TG), AST, CPK and LDH were evaluated in accordance with the test methodology outlined by the manufacturer (SPINREACT, St. Esteve de Bas, Spain) using colorimetric kits by GOD/POD/PAP, CHOD/POD/PAP, and GPO/POD/PAP methods for glucose, TCHOL, and TG analyses, respectively [30], and using a spectrophotometric kit by kinetic methods at 37 °C for ALT, CPK, and LDH activity [29].

### 2.8. Statistical Analysis

The effect of transport and slaughter was evaluated using Student’s paired t-test in order to assess the existence of significant differences between the baseline (T0) and the values after transport and slaughter (T1). The level of significance was set at *p* < 0.05. All statistical calculations were carried out using GraphPad Software (version 5, San Diego, CA, USA). Also, the correlations between circulating cortisol and 5-HT concentrations, and among hematochemical parameters were analyzed by linear regression (r), calculated by Pearson’s method. All data are expressed as mean ± standard deviation (SD).

## 3. Results

### 3.1. Circulating Cortisol and 5-HT Concentrations

**Figure 1:**
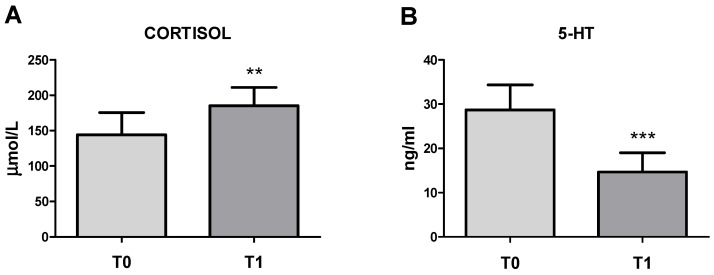
Circulating cortisol (**A**) and 5-HT concentrations (**B**) (mean ± SD) of n. 32 horses in baseline conditions (T0) and after transport and slaughter (T1). T1 vs. T0: ** *p* < 0.01 and *** *p* < 0.001.

Circulating cortisol concentrations were paired to 144.17 ± 31.33 nmol/L in baseline conditions (T0), with a significant increase (*p* < 0.01) in concentrations paired to 185.33 ± 25.64 nmol/L after transportation and slaughter (T1) (Figure 1A).

Circulating 5-HT concentrations were paired to 28.73 ± 5.64 ng/mL at T0, with a significant decrease (*p* < 0.001) in concentrations paired to 14.68 ± 4.33 ng/mL at T1 (Figure 1B). No significant correlation between circulating cortisol and 5-HT values was observed. No significant gender differences were observed.

### 3.2. 5-HT1B and 5-HT2A Serotoninergic Receptors’ Expression and IL-12 Expression

**Figure 2:**
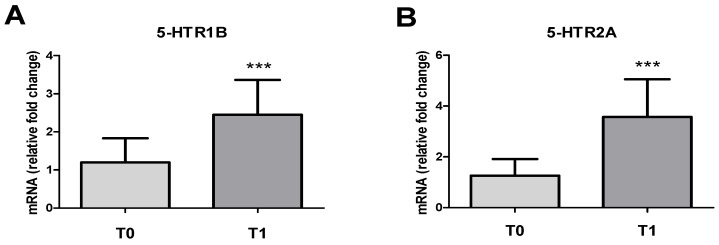
mRNA levels of 5-HT1B (**A**), and 5-HT2A (**B**) serotonin receptors in PBMCs at T0 and T1 (after transportation and slaughter). Values are expressed as the fold change with respect to the control T0 (mean ± SD; n. 32 horses). T1 vs. T0: *** *p* < 0.001.

The results of qPCR showed a significant increase (*p* < 0.001) in the expression of 5-HT1B and 5-HT2A receptors PBMCs at T1 with a fold-change of 2.4 ± 0.9 and 3.5 ± 1.5, respectively, compared to the control at T0 (Figure 2A,B). No significant gender differences were observed.

**Figure 3:**
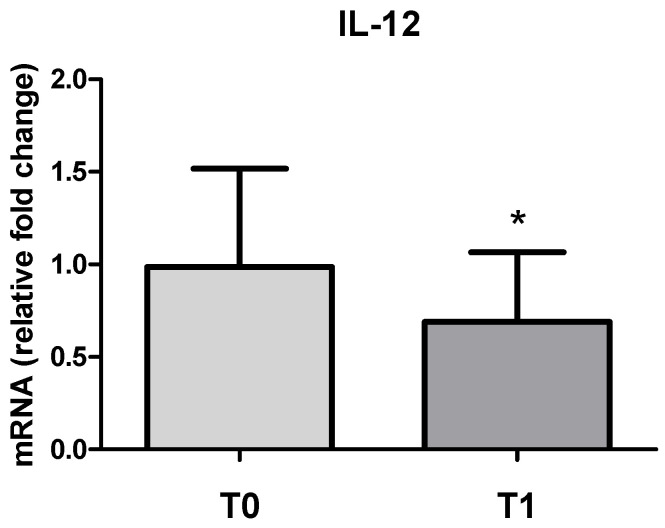
mRNA levels of IL-12 in PBMCs at T0 and T1 (after transportation and slaughter). Values are expressed as the fold change with respect to the control T0 (mean ± SD; n. 32 horses). T1 vs. T0: * *p* < 0.05.

A significant decrease in the IL-12 mRNA levels (0.7 ± 0.4 fold-change) was also observed in PBMC at T1 compared to the control at T0 (*p* < 0.05) (Figure 3). No significant gender differences were observed.

### 3.3. Hematochemical Parameters

**Figure 4:**
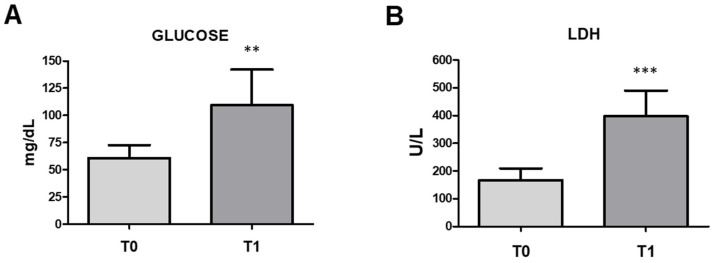
Circulating glucose (**A**) and LDH concentrations (**B**) (mean ± SD) of n. 32 horses in baseline conditions (T0) and after transport and slaughter (T1). T1 vs. T0: ** *p* < 0.01 and *** *p* < 0.001.

The distances covered during the transport and slaughter stimuli were challenging enough to induce changes in the same hematological parameters. Statistically significant differences in the values of the glucose and LDH between T0 and T1 were found (Figure 4).

Circulating glucose concentrations were paired to 60.55 ± 13.28 mg/dL in baseline conditions (T0), with a significant increase (*p* < 0.01) in concentrations paired to 111.70 ± 32.30 mg/dL after transportation and slaughter (T1). LDH activity was paired to 167.55 ± 43.21 U/L in baseline conditions (T0), with a significant increase (*p* < 0.001) in concentrations paired to 397.04 ± 93.33 U/L at (T1).

No significant differences were obtained between T0 and T1 for TG and TCHOL concentrations (Table 1), nor for AST and CPK activities (Table 1).

No significant correlations were observed among the hematochemical parameters. No significant gender differences were observed.

## 4. Discussion

The results obtained show how adrenal function, assessed through changes in circulating cortisol release, actively modulates the adaptive responses to stressful stimuli represented by short transport and superimposed slaughter conditions. This is particularly intriguing because it is not possible to distinguish between transport stress and slaughter practice effects, but the physiological response represents the sum of both stimuli.

Regardless of the suitability of the means of transport, the presence of co-specimens, and the presence of specialized personnel involved in loading and unloading procedures, transport induced an unambiguous response to the stress condition, characterized by adrenal activation. Moreover, the lack of previous transport experience in subjects used for meat production, differently from sport horses accustomed to being transported for competitions, presumably contributed to the significant increase in plasma cortisol levels, detected after transport and slaughter, also irrespective of sex, breed, and age, according to previous results observed in stallions [29].

The adrenocortical response is in accordance with the cortisol increase previously recorded both in stallions and geldings in response to excitement, transportation, and stress [31,32,33]. It is thus reasonable to hypothesize that the horse’s physical component of transport and slaughter stress stimuli may combine with the psychic one to modify the adrenocortical responses.

A poor-to-moderate relationship between the physiological response to pre-slaughter stress and meat quality was reported in pigs [34,35,36]. The moderate-to-high animal welfare experienced by pigs during transport, resulting in a low meat quality variation, may explain this poor relationship. This observation is in agreement with the findings of a review on transport research, which concluded that when transport stress is mild, meat quality is not significantly influenced as these effects are biased by rest time in lairage [37]. However, in the present study, neither the qualitative changes in the meat nor the organoleptic and inspection characteristics required by current legislation were considered. However, although the equine and porcine species are completely different, it can be assumed that even in the equine species, the repercussions on the quality of the carcass after a short road transport were almost non-existent, as shown by the results of the post-mortem examination.

Firstly, the present study was designed to establish whether stress caused by transport and slaughter routine had any effect on the physiological responses of horses. Secondly, 5-HT can provide a sensitive assessment of the stress response, modulating a wide range of immunomodulatory effects, and especially stabilizing HPA axis activity [9,11].

The different trend between circulating 5-HT and cortisol mean concentrations (T0 vs. T1 detection) found for slaughtered horses could be due to the possible inhibition or exhaustion of serotonin activity, and an activation of the adrenocortical system induced by both physical stressful stimuli and a psychological event, like transport followed by slaughter. Furthermore, the short time span between transport and slaughtering leads to the assumption of both spatial and temporal summation, with an amplified final effect of the individual stresses.

Overall, the magnitude of 5-HT decreases after the slaughter, suggesting a stimulus–response relationship and a probable exhaustion of its activity. Some studies also suggest that the physical component of short road transport stimulation may combine with the psychic slaughter one to induce 5-HT release [38,39].

Taken together, these two pieces of evidence indicate that the relative contribution provided by physical and psychic stimuli, or, more concretely, their combination, induced the adrenocortical response at the expense and in spite of 5-HT concentration changes. Moreover, the different trend between PPP 5-HT and cortisol mean concentrations confirms previous evidence in which high 5-HT concentrations seem to stabilize HPA axis activity and low concentrations could promote or facilitate stress states [9]. These data indicate that there is probably a different involvement of these markers under the same conditions, according to the different mental and physical stimuli, and the 5-HT pattern at T1 could play a consistent role through positive feedback on the HPA.

It has been reported that stress hormones’ levels can be affected by the time of sampling and other aspects of the environment [40]. Based on this evidence, is clear that all blood samples were collected under the same conditions at T0 and T1, with a similar delay to blood sampling and road transport distances. Furthermore, we reduced any potential confounding effect of age on cortisol, 5-HT concentrations, and expression of their receptors through the inclusion of only adult animals. This criterion was essential because age-related increases in 5-HT concentrations have been reported in ponies and horses [41,42].

The decrease in 5-HT concentration after transportation and slaughter stimuli could be explained by the combined effect of both, which may induce a higher degree of 5-HT storage and its successive lower plasma concentrations. Indeed, it is well known that 5-HT can be released or stored in physiological and pathological conditions as a consequence of platelet metabolism [43,44,45,46].

5-HT is also involved in modulating the immune function, inducing T-cell activation and increasing 5-HT1B and 5-HT2A receptors’ expression [47]. Furthermore, the up-regulation of 5-HT2A was also demonstrated in human monocytes that release different cytokines and chemokines after 5-HT stimulation [48]. Our data indicate that the stress to which the horses were subjected enhanced the expression levels of the serotonin receptor subtypes 5-HT1B and 5-HT2A. These results support the hypothesis that the 5-HT concentration could be higher in the initial response to stress conditions.

The detection of altered hematochemical parameters in horses subjected to transport and slaughter is consistent with the underlying acute or chronic tissues’ damage in all organisms. The highest LDH activity at T1 associated with glucose increases after transport and slaughter can be attributed to the skeletal muscle stress produced during transport by the physical activity of posture, and the consequences of the effort made during postural support to the resting barn and before slaughtering. Hence, it is possible to presume that the highest increase in LDH activity after transport and slaughter is comparable to physical exercise because it involves muscle activity. These changes in the LDH levels depend on the training state of horses [49,50]. The LDH pattern confirmed that there was a rapid increase in its activity even 24 h after transport, with its activity having been amplified by far more than previously reported from the damaging stimulus [51], and this may reflect the muscular effort of standing up during transport. LDH is an enzyme found in the myocardium, skeletal muscles, liver, kidneys, pancreas, erythrocytes, and lungs. At these sites, it is used by the body to metabolize sugar and make it available as usable energy by cells. However, the significant increase in LDH at T1 makes it more difficult to interpret the equally significant increase in glucose, almost doubled, detected at T1. It can be assumed that transport stress plus slaughter stress contributed to the disruption of homeostasis in the animals, confirming that LDH activity could represent a general indicator of the presence and severity of acute or chronic tissues’ damage. Moreover, the highest LDH activities observed after transport and slaughter could be attributed to the challenges of membrane permeability or to the increases in their synthesis or decreases in their clearance according to physical workload [50,52]. In addition, it is possible to presume that the contribution due to the adrenergic response, evocated during the first stages of transport stress, could have induced a consequent enzyme activities’ increase, representing a stronger stress-causing factor than others [53,54].

Stress exerts pleiotropic effects on the immune system, affecting the release of several cytokines, including IL-12, which exerts a broad range of biological activities. In vitro and in vivo studies have indicated that many stress factors, including glucocorticoids, may decrease IL-12 secretion [55]. Furthermore, it has been demonstrated that serotonin is able to inhibit IL-12 production by human monocytes and PBMCs stimulated by LPS [56]. Our data showed that the expression levels of IL-12 were decreased in the PBMCs of horses at T1. This might depend on the effect of the increased circulating cortisol levels that could negatively modulate the IL-12 expression in PBMCs. However, we cannot exclude that serotonin also exerts an effect on the release of this cytokine because the concentration of serotonin could change during transport and slaughter. It is possible to presume that stress induces an elevation in 5-HT synthesis at the beginning, while, subsequently, as a consequence of an increase in its reuptake, a reduction in serotonin activity could be observed.

## 5. Conclusions

The results of the present study have shown that the short road transport and pre-slaughter conditions may jointly cause a series of alterations, including the release of stress hormones, influencing horse homeostasis. Although the animals did not show visible adverse effects, our results highlighted the relevance of one health state in transported animals for slaughter purposes. The health state of animals undergoing transport procedures is a topic of growing interest nowadays as even small changes can affect animal welfare and related productivity performance after slaughter. One health represents an approach to optimize the health of humans, animals, and ecosystems by integrating these fields. The close links between human, animal and environmental health demand a proper methodological approach of the transported animal before slaughter, including good transport procedures and proper animal management that should ensure good meat quality and consumer health.

Our study suggests that appropriate transport and slaughter conditions are crucial to safeguard the welfare of horses transported over short distances for slaughter, concluding that better welfare is physiologically correlated to better meat quality. Furthermore, based on the evidence that the serotonin system is affected by road transport and slaughter, we suggest that 5-HT1B and 5-HT2A serotonin receptors’ expression could be used as a pivotal marker of stress, with potential applications for the improvement of elective protocols to observe the guidelines relating to transported horses.

## Figures and Tables

**Table 1:** Circulating hematochemical parameters (mean ± SD) of n. 32 horses in baseline conditions (T0) and after transport and slaughter (T1).

	T0	T1
TG (mg/dL)	42.6 ± 9.6	48.9 ± 7.7
TCHOL (mg/dL)	98.0 ± 7.6	76.8 ± 9.5
AST (U/L)	108.2 ± 23.0	70.0 ± 17.3
CPK (U/L)	93.3 ± 34.6	120.1 ± 25.1

## Data Availability

All relevant data are published in this article.
